# CNV Detection from Circulating Tumor DNA in Late Stage Non-Small Cell Lung Cancer Patients

**DOI:** 10.3390/genes10110926

**Published:** 2019-11-14

**Authors:** Hao Peng, Lan Lu, Zisong Zhou, Jian Liu, Dadong Zhang, Kejun Nan, Xiaochen Zhao, Fugen Li, Lei Tian, Hua Dong, Yu Yao

**Affiliations:** 1 Department of Clinical Medicine, Kunming University of Science and Technology, Yunnan 650093, China; phao9375@163.com; 2 National Cancer Center, National Clinical Research Center for Cancer, Shenzhen 518116, China; lulanlulan@163.com; 3 The Bioinformatics Department, 3D Medicines Inc., Shanghai 201114, China; zzsu1999@gmail.com (Z.Z.); fugen.li@3dmedcare.com (F.L.); 4 Department of Clinical Medicine, Guangzhou Medical University, Guangzhou 511436, China; ljian@fsyyy.com; 5 The Translational Medicine Department, 3D Medicines Inc., Shanghai 201114, China; dadong.zhang@3dmedcare.com; 6 Department of Medical Oncology, Xi’an Jiaotong University, Shaanxi 710061, China; nankj@163.com; 7 The Medical Department, 3D Medicines Inc., Shanghai 201114, China; xiaochen.zhao@3dmedcare.com; 8 Department of Thoracic Surgery Clinical Colleage, Chongqing Medical University, Chongqing 400016, China

**Keywords:** copy number variations, targeted sequencing, circulating tumor DNA, non-small cell lung cancer

## Abstract

While methods for detecting SNVs and indels in circulating tumor DNA (ctDNA) with hybridization capture-based next-generation sequencing (NGS) have been available, copy number variations (CNVs) detection is more challenging. Here, we present a method enabling CNV detection from a 150-gene panel using a very low amount of ctDNA. First, a read depth-based CNV estimation method without a paired blood sample was developed and cfDNA sequencing data from healthy people were used to build a panel of normal (PoN) model. Then, in silico and in vitro simulations were performed to define the limit of detection (LOD) for EGFR, ERBB2, and MET. Compared to the WES results of the 48 samples, the concordance rate for EGFR, ERBB2, and MET CNVs was 78%, 89.6%, and 92.4%, respectively. In another cohort profiled with the 150-gene panel from 5980 lung cancer ctDNA samples, we detected the three genes’ amplification with comparable population frequency with other cohorts. One lung adenocarcinoma patient with MET amplification detected by our method reached partial response to crizotinib. These findings show that our ctDNA CNV detection pipeline can detect CNVs with high specificity and concordance, which enables CNV calling in a non-invasive way for cancer patients when tissues are not available.

## 1. Introduction

Tumor genomic profiling plays a critical role in personalized therapy and has become a routine procedure in the diagnosis and treatment of multiple types of cancers [1,2]. Tissue biopsies sequencing is the golden standard for genomic profiling [3]. However, tumor tissues are sometimes not available in late-stage or intensively treated patients due to poor physical condition or inaccessible location of tumors. Thus, liquid biopsies sequencing becomes an alternative way of obtaining genomic information on cancer patients [4]. In recent years, circulating tumor DNA (ctDNA) has been widely used for molecular diagnosis, monitoring treatment responses, tracking clonal revolution, and detecting the emergence of cancer recurrence and drug resistance [5,6,7].

Polymerase chain reaction (PCR)-based platforms such as amplification refractory mutation system (ARMS), droplet digital PCR (ddPCR), and next-generation sequencing (NGS)-based platforms are common methods for ctDNA analysis in the clinical setting. The NGS platform possesses incomparable superiority for providing high-throughput sequencing of large numbers of genes in a single run. Moreover, almost all types of aberrations could be detected by hybrid capture-based NGS, including single nuclear variations (SNVs), short insertions/deletions (indels), gene rearrangements, and copy number variations (CNVs) [8]. Advanced technologies and algorithms have been developed to improve the sensitivity and accuracy of ctDNA variant analysis with NGS [9,10]. Methods for calling SNVs and indels in ctDNA-based sequencing have been well established. The sensitivity and specificity of detecting hot-spot mutations are both >90% [10]. However, CNV calling from ctDNA-based tests is the most challenging due to high level of biases and artifacts from limited ctDNA fraction in the whole blood circulating free DNA (cfDNA), which can be as below as 1% [4].

Comprehensive analysis of CNVs is an important component in establishing the molecular diagnosis of cancer. Aberrations in gene copy number comprising DNA amplifications and deletions represent important therapeutic targets or are associated with drug resistance and tumor biology in many cancers [11,12,13,14]. Current methods for CNV detection from ctDNA samples using target-captured sequencing require paired normal-tumor samples and are capable of detecting CNVs in prostate, gastric, bladder, breast, and lung cancer [15,16,17,18,19]. However, no systematic comparison between ctDNA and matched tissue biopsies has been performed. This is required to provide evidence to confirm the reliability of ctDNA-based CNV detection.

In this study, we used a cancer gene panel consisting of 150 genes (GP150) and developed a pipeline for detecting ctDNA CNVs without paired normal samples, utilizing multiple factory normalization and a panel of normal as an error model. Spike-in in silico simulation and in vitro cell line dilution data were generated to validate the performance and determine the limit of detection (LOD) of EGFR, ERBB2, and MET amplification. Subsequently, the concordance analysis of CNV results between ctDNA and paired tumor-normal tissues from a cohort study has been completed. Finally, CNV prevalence from ctDNA in a large independent cohort of lung cancer patients confirmed the CNV detection method and a case of non-small cell lung cancer (NSCLC) patient with MET amplification who benefitted from crizotinib treatment supported the validity of ctDNA CNV detection.

## 2. Materials and Methods 

### 2.1. Cell Culture and cfDNA Extraction

Human breast cancer cell line HCC1975 and human lung adenocarcinoma cell line NCI-H1573 were obtained from ATCC. All cells were maintained in RPMI-1640 (Hyclone, San Angelo, TX, USA) supplemented with 10% fetal bovine serum (FBS); (ThermoFisher, Waltham, MA, United States) and propagated as monolayer cultures at 37 °C in a humidified 5% CO2 incubator. The supernatant (30 mL) of cell culture medium from three 10-cm dishes was concentrated to 3 mL by an ultrafiltration concentrator Vivacell 100 (Littleton, MA, United States) for 45 min. cfDNA was extracted from a concentrate of the cell culture medium supernatant using a QIAamp Circulating Nucleic Acid Kit (Qiagen, Dusseldorf, Germany) according to the manufacturer’s instructions.

### 2.2. Sample Collection

Ten plasma samples from healthy people were selected as a reference data set to build the panel of normal controls. Approximately 48 NSCLCs with matched tumor tissues and blood samples were collected between August 2015 and January 2018 and met the following criteria: (1) Stages IIIB or IV patients; (2) blood samples were collected before surgery of tumor samples or within 14 days apart from tumor biopsy samples; and (3) tumor tissues were determined based on the percentage of tumor cells by H&E staining. Only samples with a tumor cell percentage >20% were included in this study. All patients provided specimens with written informed consent. All participants gave their informed consent before they participated in the study. Another cohort of 5980 NSCLC cfDNA samples from Chinese patients were collected between July 2017 and September 2019 and were tested in CAP and CLIA accredited laboratory (3D Medicines, Shanghai, China) for hybrid capture, followed by next-generation sequencing of the GP150 panel. The study was conducted in accordance with the Declaration of Helsinki, and the protocol was approved by the Ethics Committee of Xi’an Jiaotong University. 

### 2.3. DNA Extraction

Blood was centrifuged in STRECK tubes at 1600 g for 20 min at room temperature to separate plasma. Then, the plasma layer was carefully transferred to a new 1.5-mL Eppendorf tube, followed by room temperature centrifugation at 16,000 g for 10 min to remove the residual cells and debris. The buffy coat was then transferred to a new tube for genomic DNA (gDNA) extraction. Afterward, gDNA from tumor FFPE tissues and white blood cells were extracted by the DNeasy Tissue or Blood Kit (Qiagen, Dusseldorf, Germany) following standard protocols and then fragmented to a size ranging from 200 bp to 400 bp using Covaris S2 Sonolab (Covaris, Woburn, MA, USA). The QiAamp Circulating Nucleic Acid Kit (Qiagen, Dusseldorf, Germany) was used to extract cfDNA from plasma. DNA concentrations were determined using a Qubit dsDNA HS Assay Kit (Life Technologies, Carlsbad, CA, USA).

### 2.4. Library Preparation, Target Capture, and DNA Sequencing

gDNA libraries were established by KAPA Hyper Prep Kit (KAPA Biosystems) according to the manual. The cfDNA libraries were prepared using an Accel-NGS 2S Plus DNA Library Kit (SWIFT) with unique identifiers (UIDs, also called barcoding technology) to tag individual DNA molecules. The concentration and size distributions of libraries were respectively analyzed using Qubit and Caliper.

The pooled DNAs were mixed with 2 μL of DNA blocker (Integrated DNA Technologies) and 5 μL of human Cot-1 DNA (Invitrogen), and then dried using a vacuum concentrator (Thermo Fisher). The dried mixture was dissolved in a 15 μL of hybridization buffer supplied by the hybridization of xGen Lockdown Probes Kit (Integrated DNA Technologies), and thereafter IDT xGen Human Exome Research Panel kit was used to capture targeted DNAs for FFPE gDNA following the standard protocol. For plasma cfDNA library, we used a customized DNA probes in 150 genes (GP150) with unique identification (UID) indexed capturing-based sequencing (UC-Seq) method [20].

The captured libraries for FFPE gDNA were loaded into the HiSeq X (illumina) to 150-bp paired-end sequencing, and the captured libraries for plasma cfDNA were loaded into the NextSeq500 (illumina) to run 75-bp paired-end sequencing according to the manufacturer’s instructions.

### 2.5. ctDNA CNV Calling Pipeline

Raw fastq files were aligned using bwa-mem against reference genome hg19 and raw read depth were counted probe by probe. Read depth was normalized by the median depth of all probes. A combined model of GC content, probe overlap score, and mapping ability was applied to read count data to further reduce bias. Then we used cfDNA sequencing data from 10 healthy people to build a panel of normal (PoN) model. Tumor samples’ read depths were further normalized by PoN model. We used a traditional circular binary segmentation (CBS) method to segment the resulted log ratios. The standard deviation (SD) of all segment level log-ratios was calculated for each sample and segments with log ratio above 3*SD were treated as amplification. Because the ploidy of ctDNA is hard to estimate accurately and ctDNA content in cfDNA is usually low, we did not estimate genomic deletion in this pipeline.

Calling absolute copy number (ABCN) depends on tumor fraction estimation from cfDNA. We used insert size-based method to estimate ctDNA fraction in ctDNA. It has been reported that ctDNA molecules from tumor cells are shorter than the cell-free molecules from normal cells [20,21]. We inferred the ctDNA fraction by the distribution difference between ctDNA and normal cfDNA. Proportion of cfDNA fragments below 150 bp was referred as the proportion of ctDNA faction in total cfDNA [22].

For sample i, gene j, with tumor purity p, log2ratio is calculated by formula (1):

(1) $$log2Ratio\left[i,j\right]=log2\frac{ABCN\left[i,j\right]*p+2*\left(1-p\right)}{2}$$

Thus we could infer the ABCN by formula (2):

(2) $$ABCN\left[i,j\right]=\frac{2*\left(2^{log2Ratio\left[i,j\right]}+p-1\right)}{p}$$

The pipeline could be accessed via Github link: https://github.com/3dmed-bioi/btcnv. The raw sequencing data has been submitted to NCBI SRA database, here is the accession link: https://www.ncbi.nlm.nih.gov/bioproject/PRJNA557300.

### 2.6. WES CNV Calling Pipeline for Tumor-Normal Sample Pairs

Raw data (fastq files) with paired samples (FFPE and its normal control) were aligned to the human genome (hg19) using BWA aligner v0.7.12. PCR duplicate reads were removed and sequence metric collection were done using Picard: https://github.com/broadinstitute/picard/releases/tag/1.130. Copy number variation analysis was performed using FACETS V0.5.6: https://github.com/mskcc/facets [23]. Tumor fraction was estimated by FACETS from WES tumor samples’ data and absolute copy number (ABCN) were called based on the same formula (2) above.

## 3. Results

### 3.1. ctDNA CNV Detection Assay and Pipeline Development

**Figure 1:**
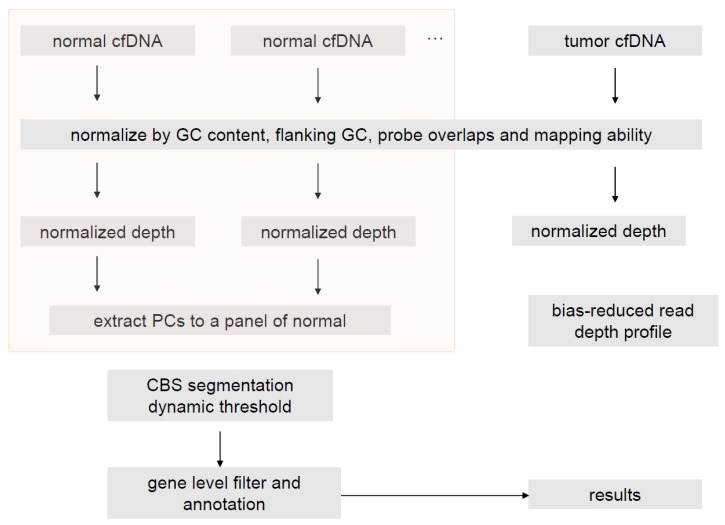
Flowchart of the copy number variation (CNV) calling pipeline.

We have designed a 150-gene panel by IDT capture method that encompasses full exons of the 150 genes and some extra intronic probes for fusion calling. The assay development has been validated in a CAP-accredited lab in 3DMed and the concordance between ctDNA SNV calling and blood tumor mutation load (bTMB) has been published [20,24]. Here a coverage-based copy number estimation method without paired blood samples was developed and the main workflow is shown is Figure 1. Absolute copy number (ABCN) was estimated by gene log2ratio and tumor fraction estimation from cfDNA.

### 3.2. In Silico Simulation and In Vitro Dilution Validation to Train the Pipeline

**Figure 2:**
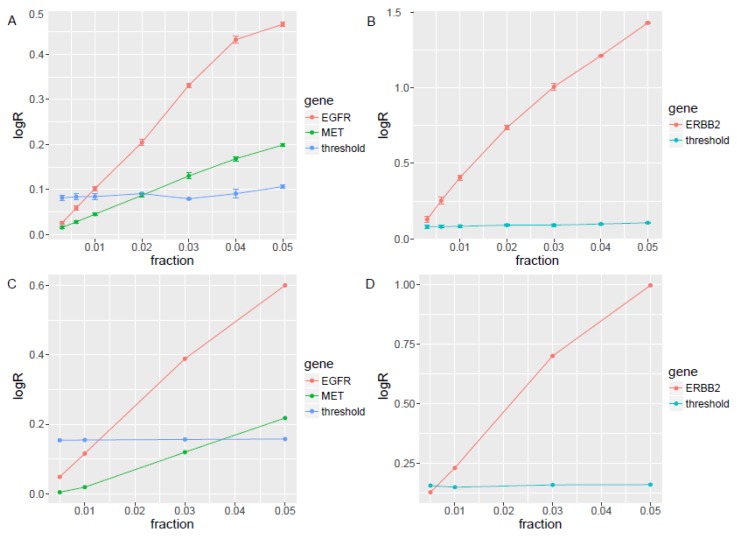
The EGFR, MET, and ERBB2 limits of detection (LODs) in simulated samples and in vitro spike-in samples. (**A**) In silico simulation of NCI-H1573 cell line data with EGFR, and MET amplification into three sets of four normal cfDNA samples sequenced with the same panel in a series of 5%, 4%, 3%, 2%, 1%, 0.6%, and 0.3% to reach a sequencing depth of 10,000x to define the LOD for EGFR and MET. (**B**) In silico simulation of HCC1954 cell line data with ERBB2 amplification into three sets of four normal cfDNA samples sequenced with the same panel in a series of 5%, 4%, 3%, 2%, 1%, 0.6%, and 0.3% to reach a sequencing depth of 10,000x to define the LOD for ERBB2. (**C**) In vitro experiments dilution NCI-H1573 cell line’ cfDNA described above with another set of four normal cfDNAs to tumor fractions of 5%, 3%, 1%, and 0.5% by in vitro experiments to re-determine the LOD for EGFR and MET. (**D**) In vitro experiments dilution HCC1954 cell line’ cfDNA described above with another set of four normal cfDNAs to tumor fractions of 5%, 3%, 1%, and 0.5% by in vitro experiments to re-determine the LOD for ERBB2.

Two cell lines, NCI-H1573 and HCC1954, with important CNVs in clinical practice, including ERBB2 (HCC1954), EGFR, and MET (NCI-H1573), were used for technical validation. The copy number of MET and EGFR is 13 and 20 in NCI-H1573 respectively, and ERBB2 is 60 in HCC1954 from the CCLE database [25,26]. We generated sequence files by down-sampling the CNV positive sequences and spiking into three sets of four normal cfDNA samples sequenced with the same panel in a series of 5%, 4%, 3%, 2%, 1%, 0.6%, and 0.3% to reach a sequencing depth of 10,000x. The LOD was about 0.3% for ERBB2 (2.2 copies), 3% for MET (2.5 copies), and 1% for EGFR (2.2 copies) (Figure 2A,B). The small standard error assures high reproducibility with different sets of Panel of Normal.

We diluted the same cell lines’ cfDNA described above with another set of four normal cfDNAs to tumor fractions of 5%, 3%, 1%, and 0.5% by in vitro experiments to re-determine the LOD. The LOD based on in vitro dilution was about 1% (2.5 copies) for ERBB2, 3% (2.5 copies) for EGFR, and 5% (2.5 copies) for MET (Figure 2C,D), which is slightly higher than these in silico simulation. Our ctDNA CNV experimental validation data also showed that the target CNV can be detected when the ctDNA ratio is 0.3–3% in silico and 1%~5% in vitro, which is in line with or better than the reference data [27,28].

### 3.3. Clinical Sample Validation

To further validate the performance of the ctDNA pipeline, we performed the profiling of paired tumor-normal WES and matched cfDNA with 150-gene panel in a cohort with 48 small cell lung cancer patients. Patient demographic and clinical characteristics of this cohort is shown in Supplementary Table S1. The absolute copy number of the WES was determined using FACETS software with default parameters [23], and gene level copy number was determined by overlapping CNV segments with RefSeq gene annotations. For EGFR, ERBB2, and MET gene amplification, we set the absolute copy number ≥6 as amplification for WES with tumor and normal pairs and log2 ratio ≥ 3-fold standard derivation (SD) as amplification for the ctDNA compared with a panel of normals. With the WES CNV results as a reference standard, the accuracy or concordance rate for EGFR, ERBB2, and MET CNV calling for ctDNA was 78%, 89.6%, and 92.4%, respectively. The detection sensitivity for EGFR, ERBB2, and MET in ctDNA was 35%, 37.5%, and 40%, respectively, and the specificity was 100% for all the three genes.

**Figure 3:**
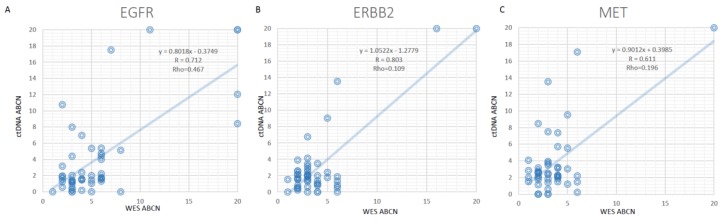
The absolute copy number (ABCN) correlation of EGFR, ERBB2, and MET between WES and ctDNA. (**A**) for EGFR, (**B**) for ERBB2, and (**C**) for MET.

The absolute copy number (ABCN) was estimated for ctDNA based on both DNA fragment distribution and average variant allelic frequency. Then the ABCNs of EGFR, ERBB2, and MET between tissue WES and cfDNA were positively correlated (Figure 3). Although our method was trained on only three genes, it could be extended to other genes in the panel. When we estimate the overall performance for 150 genes on the panel, using tissue WES as a standard. The overall sensitivity was 65% when absolute copy number was >6, and 71% when absolute copy number was >13 (Supplementary Figure S1).

### 3.4. An Independent Clinical Cohort Re-Confirmed the Pipeline

**Figure 4:**
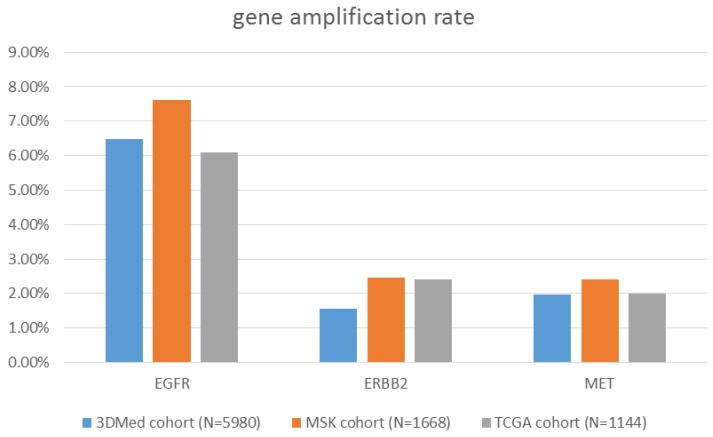
Comparison of EGFR, ERBB2, and MET gene amplification rate from a large cohort of lung cancer patient circulating tumor DNA (ctDNA) profiling (N = 5980) with two large cohorts with tissue profiling: MSK (N = 1668) and TCGA (N = 1144).

For further clinical validation, we profiled 5980 ctDNA samples from the real-world NSCLC cohort with the 150-gene panel. The amplifications involving EGFR, HER2, and MET were detected in 387 (6.47%), 93 (1.56%), and 118 (1.97%) samples, respectively. We compared the gene amplification percentage with two public cohorts from cBioPortal (www.cbioportal.org): MSKCC-IMPACT clinical sequencing cohort for NSCLC, including 1668 tissue samples, and TCGA NSCLC cohort, including 1144 tissue samples. CNVs detected in these two cohorts were 7.62% and 6.10% for EGFR, 2.46% and 2.40% for HER2, and 2.40% and 2.01% for MET, respectively [29,30]. The comparison results are shown in Figure 4. The difference of recurrence of EGFR/ERBB2/MET is probably due to the different stage of tissues, as the majority data of MSKCC-IMPACT is from late-stage biopsy tissues; while TCGA NSCLC cohort, from early-stage lung cancer primary tumor tissues. The amplification ratio of EGFR/ERBB2/MET in ctDNA samples is slightly lower, which could be contributed by low sensitivity of CNV detection in ctDNA. Although age and sex were matched for the three cohort, the presence of druggable mutation is not matched due to the ethnicity and cancer stages: the cohort of 3DMed cfDNA samples are from Chinese late stage NSCLC patients, which includes higher EGFR mutation rate and lower KRAS rate; the cohort of MSKCC-IMPACT samples are mostly US late stage NSCLC patients and TCGA NSCLC cohort is mostly US early stage NSCLC patients. As large Chinese NSCLC cohort with cfDNA profiling data is not available, we use these well-known public datasets as reference for comparison.

### 3.5. Clinical Case Demonstrated This Patient with MET Amplification Was Response to Crizotinib

**Figure 5:**
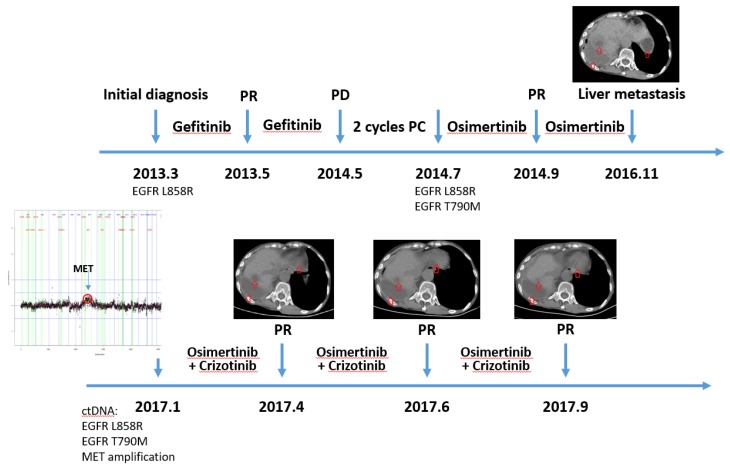
Timeline of clonal evolution and treatment management for an MET amplification patient.

A female patient with lung adenocarcinoma accepted gefitinib as first-line therapy because of EGFR L858R mutation and achieved partial response. One year later, she progressed with extrathoracic metastasis, and gained EGFR T790M mutation in her metastatic lesion. She took medication of osimertinib and reached partial response again. After two-year treatment with osmertinib, she developed multiple liver metastasis. A tissue biopsy was not feasible because of her poor condition and the high risk of re-biopsy. Thus, plasma ctDNA was profiled with a GP150 panel. MET amplification was detected with a copy number of 10. MET amplification was considered as the mechanism of acquired resistance to osimertinib [31]. She was treated with crizotinib and reached partial response. Her disease remained under control during the latest follow-up. Patient treatment history and follow-up with accompanying images are shown in Figure 5. 

## 4. Discussion

Detection of somatic copy number variations from ctDNA samples using targeted sequencing is very challenging. In a 2017 study by Chae et al. [19], the comparison between tissue profiling by FoundationOne and ctDNA profiling by Guardant360 from 45 patients demonstrated the concordance of CNV detection (total 86 CNVs) was only 3.5%. Another study in 45 prostate cancer patients [15] showed the concordance was 48.9% (22/45) in cfDNA vs. tissue, which is much better because it required the samples had sufficiently high ctDNA fraction (>35%). For those samples, there was a very high correlation between the coverage log ratios of ctDNA and matched tissue biopsies (median R^2^ = 0.76). Across the clinically informative mCRPC driver genes AR, BRCA2, ATM, PTEN, PIK3CA, PIK3CB, PIK3R1, TP53, and RB1, the authors observed a concordance of 88.9% for individual gene CNV between evaluable ctDNA and tissue biopsies. However, another report showed that tumor-derived cfDNA reach 10% in only 33.3% of prostate cancer patients, and above 35% in 12% of patients [28]. In a Squamous Lung Cancer Clinical Cohort, patients with high tumor fraction (>25%) in the plasma, CNV patterns in cfDNA and tissue generally showed high concordance, with Pearson correlation values greater than 0.75. Pearson correlation values were low (0.3–0.6) when cfDNA tumor fraction was less than 25%, and below 0.2 when cfDNA tumor fraction was 0% [32].

In our study, we have also performed head-to-head comparison of EGFR, ERBB2, and MET gene amplifications between tissue WES and ctDNA in 48 lung cancer patients. The concordance of EGFR, ERBB2, and MET CNV was 78%, 89.6%, and 92.4%, respectively. The detection sensitivity for EGFR, ERBB2, and MET was 35%, 37.5%, and 40%, respectively, and the specificity was 100% for all the three genes. The performance was better than the previously reported datasets in breast and prostate cancer and comparable to theoretical simulation [15,19,27,28].

Molparia et al. assessed circulating CNV detection for cancer screening based on theoretical simulation data, which showed that CNV calling accuracy is correlated with cancer types, because the performance is affected by these factors such as cfDNA release content to the circulating blood, CNV calling resolution and algorithm [27]. However, Molparia et al. did not evaluate and discuss the possible factors from NGS experiments. The variables affecting CNV calling include (1) ctDNA concentration in cfDNA; (2) biases caused by the experiment procedures such as PCR and library preparing steps, targeted capture, and sequencing procedure; and (3) the heterogeneity and clonal evolution. Further improvement of CNV detection in ctDNA is possible. ctDNA enrichment by shorter fragment size [22] to increase ctDNA concentration of cfDNA could overcome the low ctDNA fraction to affect CNV detection. The normalization based on GC content could reduce bias. We used a panel of cfDNA from healthy volunteers to build the background model, which could remove some bias from experiments and platform, and enhance the accuracy of CNV detection in ctDNA.

Our ctDNA CNV pipeline demonstrated the ability to identify ctDNA CNV in late stage non-small cell lung cancer patients where tissue biopsy is usually not available. In this study, we largely focus on the EGFR/ERBB2/MET gene amplification in late stage NSCLC, EGFR, ERBB2, and MET amplification are the major resistant mechanism for EGFR T790M mutation patients, account for 39.5% in Chabon, J.J et al’s paper [18]. In the internal cohort of NSCLC cfDNA samples, the major druggable mutation rate was as follows: EGFR, ERBB2, and MET amplification account for 19.7% in EGFR driver mutation positive patients, and account for only 1.92% in EGFR driver mutation negative patients, respectively, which also suggest that EGFR, ERBB2 and MET amplification were enriched in EGFR TKI resistant patients (Odds Ratio: 12.58; Fisher exact test *P*-value: < 2.2 × 10^16^). Our ctDNA CNV pipeline application would highly benefit in late stage non-small cell lung cancer patients when tissue biopsy is not available, especially in EGFR TKI resistant patients, where EGFR, ERBB2 and MET can account for 20%~40% patient population and no effective liquid biopsy assay to identify the resistant mechanism and following treatment decision. Additionally, our pipeline can be extended to other cancer types. However, clinical validation should be carefully performed before this assay is applied to a clinical laboratory.

## 5. Conclusions

CNV detection from ctDNA based on targeted sequencing has been challenging due to limited ctDNA fractions in blood circulation. To improve the clinical utility of CNV detection, we developed a pipeline based on a panel of normal as an error model and ctDNA estimation from DNA fragment distribution. We have demonstrated that the CNV pipeline can detect EGFR, ERBB2, and MET amplifications from ctDNA samples, which are highly concordant with these from the corresponding tissue-based WES. An independent cohort was used for further clinical validation of gene amplification rates from real-world NSCLC patients. Although actionable gene amplification could not be detected in ctDNA every time, we presented a clinical case with MET amplification detected in ctDNA and this patient was response to crizotinib. In summary, our proof-of-concept study both technically and clinically validated the ctDNA CNV detection pipeline, which enables CNV calling in a noninvasive way for late stage NSCLC patients when tissues are not available.

## Figures and Tables

**Figure A1:**
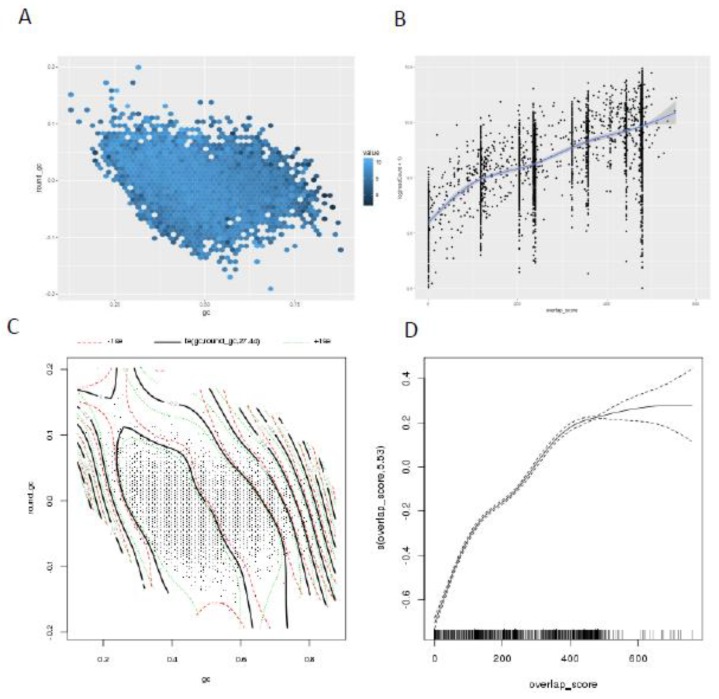
Normalization of different coverage bias. (**A**) The implications of GC and flanking GC. (**B**) The implications of probe overlap score. (**C**,**D**) The integrated scheme of them in a real example.

**Figure A2:**
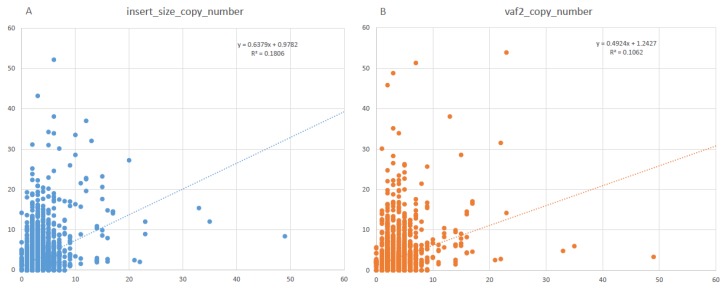
The correlation of WES absolute copy number and ctDNA absolute copy number estimation by two methods: (**A**) insert size; (**B**) variant allele frequency.

## Supplementary Materials

The following are available online at https://www.mdpi.com/2073-4425/10/11/926/s1, Figure S1. The total sensitivity and specificity curve for absolute copy number based on different WES absolute copy number gain cut-off from 6 to 13; Table S1. Patient demographic and clinical characteristics of the 48 paired sample cohort.

## Appendix A

There are five major steps included in the ctDNA CNV calling pipeline,

Step 1. Preprocess depth of reads per probe. Raw reads were aligned using bwa mem against reference genome hg19; raw read depth were counted in a base within a probe.

Step 2. Normalization and bias correction. Outliers in read depth were first removed, then read depth was normalized by the median depth of all probes.

Step 3. A combined model of GC content, probe overlap score, and mapping ability was applied to read count data to further reduce bias. The conventional GC bias correction is a local polynomial regression fitting (LOESS) [33], while we used spline fitting model to correct GC content bias. Not only the target region GC content, but also the flanking region GC content will also influence the probe coverage, as shown in Figure A1. For overlapped probes, we assumed that the capability of capture is the same when the probe fully overlaps with the DNA segment, and linearly decreases as the length of overlap decreases, and we applied this assumption to the whole probe set in the panel. As we assumed, probe level depth increases near-linearly with the overlap score and saturates when the score is high enough. We also modeled the overlap score as a component in the model using spline fitting.

Step 4. Error correction by a panel of normal. After pervious normalization and bias corrections, some biases of unknown region were still noticeable, so we introduced a panel of normal strategy to further refine the data.

Step 5. Segmentation and adaptive threshold. After normal segmentation, we used a traditional circular binary segmentation (CBS) method to segment the resulted log ratios. The standard deviation (SD) of all segment level log-ratios was then calculated for each sample, and segments with log ratio above 3*SD were treated as amplification.

Calling absolute CNV depends on tumor fraction estimation from cfDNA. We used two methods to estimate tumor fraction in cfDNA. The first is the variant allele frequency-based method. Variant calling from cfDNA was introduced in our previous paper [20]. ctDNA fraction was estimated as two-fold of the average variant allele frequency in the sample. ctDNA fractions were estimated as 0 when no variants were called. The other estimation method we used is the DNA fragment distribution. It has been reported that ctDNA molecules from tumor cells are shorter than the cell-free molecules from normal cells [20,21]. We inferred the ctDNA fraction by the distribution difference between ctDNA and normal cfDNA. We compared the two approaches with the WES CNV data, and the results showed that ABCN estimated by DNA fragment distribution correlates better than the average VAF as shown in Figure A2.
