# Supplementary material for: CNV Detection from Circulating Tumor DNA in Late Stage Non-Small Cell Lung Cancer Patients
Source: Genes (Basel). 2019 Nov 14;10(11):926. doi: 10.3390/genes10110926 (PMC6895974; doi:10.3390/genes10110926)
Supplement: Supplementary file 1 [file genes-10-00926-s001.pdf]

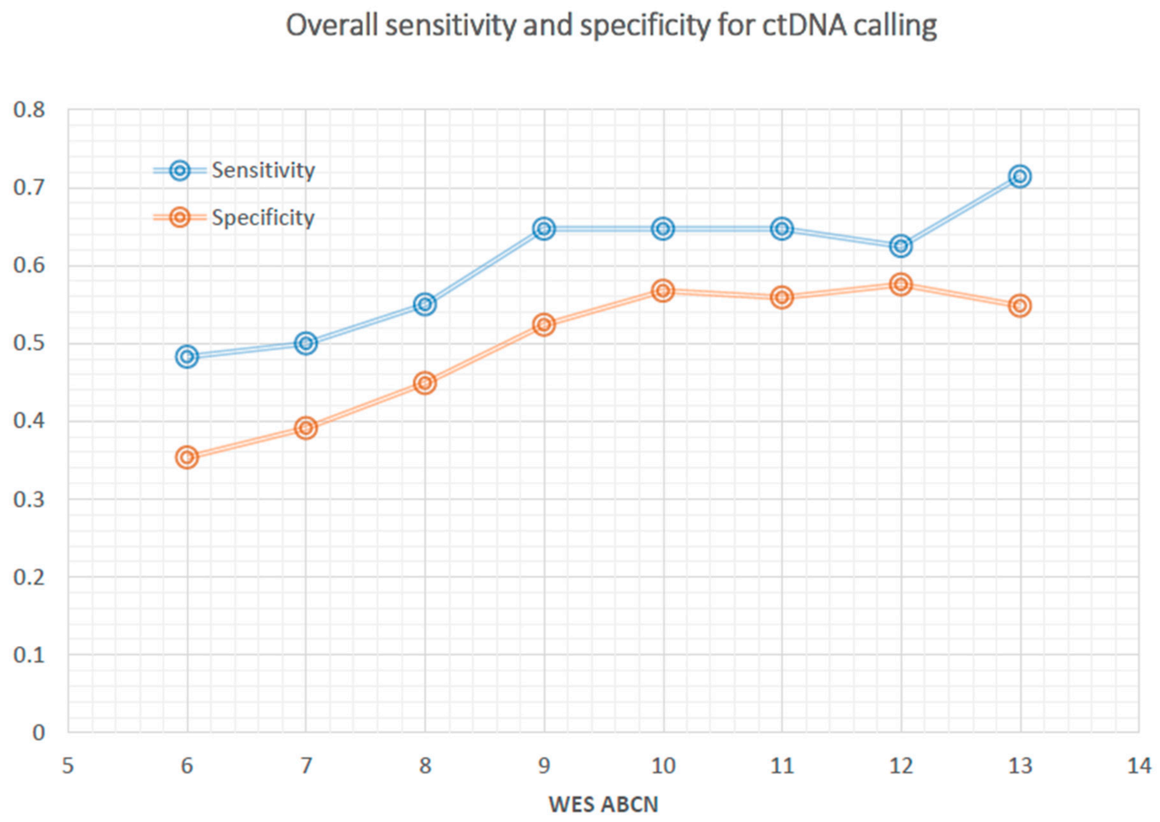

Figure S1. The total sensitivity and specificity curve for absolute copy number based on different WES absolute copy number gain cut-off from 6 to 13.

Table S1. Patient demographic and clinical characteristics of the 48 paired sample cohort.

Sample_ID	Gender	Age	TNM	Tumor sampling date	Blood sampling date	Tumor sample type	Tumor percentage
10528S01	Male	69	IV	13-Oct-2017	17-Oct-2017	Biopsy	0.7
10763S01	Male	64	IIIb	26-Oct-2017	26-Oct-2017	Biopsy	0.3
10839S02	Male	54	IV	23-Oct-2017	31-Oct-2017	Biopsy	0.25
11455S01X1	Male	80	IV	21-Nov-2017	24-Nov-2017	Biopsy	0.3
11590S01X1	Male	61	IV	21-Nov-2017	30-Nov-2017	Biopsy	0.2
12328S01	Male	67	IV	21-Dec-2017	25-Dec-2017	Biopsy	0.3
12426S01	Male	63	IV	27-Dec-2017	2-Jan-2018	Biopsy	0.75
12972S02	Male	69	IV	24-Jan-2018	23-Jan-2018	Biopsy	0.5
ct10416S01	Female	57	IV	19-Aug-2016	19-Aug-2016	Biopsy	0.8
ct10423S01	Male	58	IV	11-Aug-2016	16-Aug-2016	Biopsy	0.5
ct10444S01	Female	46	IV	18-Jul-2016	18-Jul-2016	Biopsy	0.7
ct20004S01	Female	68	IV	16-Jun-2016	21-Jun-2016	Biopsy	0.3
ct20119S01	Male	75	IV	6-Jul-2016	7-Jul-2016	Biopsy	0.6
ct20145S01	Female	67	IV	17-Aug-2016	17-Aug-2016	Biopsy	0.8
ct20164S01	Male	61	IIIb	6-Sep-2016	9-Sep-2016	Biopsy	0.8
ct20183S01	Female	67	IV	6-Jul-2016	5-Jul-2016	Thoracoscopy	0.5
ct20232S01	Female	53	IV	4-Jul-2016	4-Jul-2016	Biopsy	0.3
ct20236S01	Female	57	IIIb	22-Jul-2016	29-Jul-2016	Biopsy	0.8
ct20237S01	Female	69	IIIb	13-Jul-2016	13-Jul-2016	Biopsy	0.4

ct20239S01	Male	26	IV	1-Jul-2016	1-Jul-2016	Biopsy	0.75
ct20241S01	Male	55	IIIb	7-Jul-2016	7-Jul-2016	Biopsy	0.4
ct20246S01	Female	61	IV	13-Jul-2016	12-Jul-2016	Surgery	0.4
ct20247S01	Male	83	IV	1-Sep-2016	9-Sep-2016	Biopsy	0.7
ct20262S01	Male	77	IIIb	8-Aug-2016	11-Aug-2016	Biopsy	0.6
ct20280S01	Female	56	IIIB	22-Jul-2016	22-Jul-2016	Biopsy	0.3
ct20295S01	Female	52	IV	18-Jul-2016	18-Jul-2016	Biopsy	0.4
ct20299S01	Male	62	IIIb	1-Jul-2016	5-Jul-2016	Biopsy	0.7
ct20320S01	Female	57	IV	4-Aug-2016	9-Aug-2016	Biopsy	0.3
ct20394S01	Female	50	IV	30-Aug-2016	31-Aug-2016	Biopsy	0.3
ct20395S01	Female	27	IV	15-Aug-2016	25-Aug-2016	Biopsy	0.2
ct20401S01	Male	68	IV	2-Sep-2016	2-Sep-2016	Biopsy	0.4
ct20402S01	Female	77	IIIB	4-Sep-2016	1-Sep-2016	Surgery	0.5
ct20410S01	Male	74	IV	1-Sep-2016	12-Sep-2016	Biopsy	0.4
ct20414S01	Male	65	IV	14-Sep-2016	14-Sep-2016	Fiberoptic bronchoscopy	0.4
ct20416S01	Male	59	IV	20-Sep-2016	20-Sep-2016	Biopsy	0.4
101S01	Male	62	IV	12-Feb-2015	25-Feb-2015	Biopsy	0.8
103S01	Male	32	IV	17-Sep-2015	28-Sep-2015	Biopsy	0.9
108S01	Male	73	IV	10-Jun-2015	21-Jun-2015	Biopsy	0.3
109S01	Male	74	IV	27-Jul-2015	4-Aug-2015	Biopsy	0.2
110S01	Male	52	IV	20-Jul-2015	16-Jul-2015	Surgery	0.2
118S01	Male	65	IV	13-Aug-2015	13-Aug-2015	Biopsy	0.3
122S01	Male	52	IV	18-Dec-2015	18-Dec-2015	Unknown	0.6
127S01	Male	54	IV	10-Oct-2015	16-Oct-2015	Biopsy	0.6
129S01	Male	52	IV	30-Nov-2015	11-Dec-2015	Biopsy	0.4
132S01	Male	56	IV	21-May-2015	28-May-2015	Biopsy	0.7
134S01	Male	50	IV	2-Jul-2015	9-Jul-2015	Biopsy	0.5
135S01	Male	39	IV	22-Jul-2015	24-Jul-2015	Biopsy	0.5
138S01	Male	43	IV	16-Jul-2015	16-Jul-2015	Surgery	0.85
